# Fluvastatin in the therapy of acute coronary syndrome: Rationale and design of a multicenter, randomized, double-blind, placebo-controlled trial (The FACS Trial)[ISRCTN81331696]

**DOI:** 10.1186/1468-6708-6-4

**Published:** 2005-03-24

**Authors:** Petr Ostadal, David Alan, Petr Hajek, Jiri Vejvoda, Martin Mates, Peter Blasko, Josef Veselka, Milan Kvapil, Jiri Kettner, Martin Wiendl, Ondrej Aschermann, Josef Slaby, Eduard Nemecek, Frantisek Holm, Marek Rac, Milan Macek, Jana Cepova

**Affiliations:** 1 Department of Cardiology, University Hospital Motol and Charles University, 2nd Faculty of Medicine, Prague, Czech Republic; 2 Department of Internal Medicine, University Hospital Motol and Charles University, 2nd Faculty of Medicine, Prague, Czech Republic; 3 Department of Cardiology, Institute for Clinical and Experimental Medicine, Prague, Czech Republic; 4 Department of Cardiology, Na Homolce Hospital, Prague, Czech Republic; 5 Department of Medicine, Hospital Kolin, Kolin, Czech Republic; 6 1st Department of Medicine, Hospital Na Frantisku, Prague, Czech Republic; 7 Department of Cardiology, Regional Hospital Liberec, Liberec, Czech Republic; 8 Internal Medicine & Clinical Pharmacology Department, Faculty Hospital Nitra, Nitra, Slovakia; 9 Institute of Biology and Medical Genetics, University Hospital Motol and Charles University, 2nd Faculty of Medicine, Prague, Czech Republic; 10 Department of Clinical Biochemistry and Pathobiochemistry, University Hospital Motol and Charles University, 2nd Faculty of Medicine, Prague, Czech Republic

**Keywords:** statin, fluvastatin, acute coronary syndrome, C-reactive protein, interleukin 6, pregnancy-associated plasma protein A

## Abstract

**Background:**

Activation of inflammatory pathways plays an important contributory role in coronary plaque instability and subsequent rupture, which can lead to the development of acute coronary syndrome (ACS). Elevated levels of serum inflammatory markers such as C-reactive protein (CRP) represent independent risk factors for further cardiovascular events. Recent evidence indicates that in addition to lowering cholesterol levels, statins also decrease levels of inflammatory markers. Previous controlled clinical trials reporting the positive effects of statins in participants with ACS were designed for very early secondary prevention. To our knowledge, no controlled trials have evaluated the potential benefits of statin therapy, beginning immediately at the time of hospital admission. A previous pilot study performed by our group focused on early initiation of cerivastatin therapy. We demonstrated a highly significant reduction in levels of inflammatory markers (CRP and interleukin-6). Based on these preliminary findings, we are conducting a clinical trial to evaluate the efficacy of another statin, fluvastatin, as an early intervention in patients with ACS.

**Methods:**

The FACS-trial (Fluvastatin in the therapy of Acute Coronary Syndrome) is a multicenter, randomized, double-blind, placebo-controlled study evaluating the effects of fluvastatin therapy initiated at the time of hospital admission. The study will enroll 1,000 participants admitted to hospital for ACS (both with and without ST elevation). The primary endpoint for the study is the influence of fluvastatin therapy on levels of inflammatory markers (CRP and interleukin-6) and on pregnancy associated plasma protein A (PAPP-A). A combined secondary endpoint is 30-day and one-year occurrence of death, nonfatal myocardial infarction, recurrent symptomatic ischemia, urgent revascularization, and cardiac arrest.

**Conclusion:**

The primary objective of the FACS trial is to demonstrate that statin therapy, when started immediately after hospital admission for ACS, results in reduction of inflammation and improvement of prognosis. This study may contribute to new knowledge regarding therapeutic strategies for patients suffering from ACS and may offer additional clinical indications for the use of statins.

## Background

During the past decade, inflammation has often been cited as a major factor in the pathogenesis of atherosclerosis and its clinical sequelae, including ischemic heart disease. It was found that traditional risk factors such as hypertension, hypercholesterolemia, diabetes, and smoking could not fully account for the development of coronary stenosis in all patients suffering from ischemic heart disease. Intensive study of the pathogenesis of coronary plaque development and rupture led to the hypothesis that inflammatory factors contribute to this process. For example, T-lymphocytes and monocytes/macrophages have been repeatedly identified in plaque lesions; elevated levels of acute phase proteins (C-reactive protein, serum amyloid A, fibrinogen), cytokines (interleukin 1, interleukin 6, interleukin 8, tumor necrosis factor), and adhesive molecules (ICAM-1) correlate with the worse prognoses in patients with ischemic heart disease [1-5]. Furthermore, the increased level of C-reactive protein (CRP) is now widely recognized as being an independent risk factor for a higher incidence of non-fatal and fatal coronary events in patients with chronic ischemic heart disease and acute coronary syndromes [6-9].

Activation of the immune reaction in acute ischemic heart disease likely derives from: (i) pathological events occurring in the arterial wall where the lesion develops, leading to plaque rupture and the subsequent clinical consequences and (ii) myocardial necrosis, which triggers processes involved in removal of the necrotic mass and replacement with scar tissue. Whereas activation of plaque inflammation (as noted above) serves as a marker for plaque instability, elevation of inflammatory factors from the second source correlates with the extent of myocardial necrosis.

Statins, 3-hydroxy-3-methylglutaryl coenzyme A (HMG-CoA) reductase inhibitors, lower cholesterol levels by decreasing the production of low-density lipoproteins (LDL) and up-regulating the expression of the LDL-receptor. These drugs are widely used in patients with hypercholesterolemia for primary and secondary prevention of coronary artery disease because of their efficacy in reducing cardiovascular morbidity and mortality [10-12]. Surprisingly, statin therapy also improves prognosis in patients with normal or low cholesterol levels[13]. Evaluation of the non-lipid effects of statins reveals a possible beneficial effect mediated by the reduction of inflammatory markers, namely, CRP. This effect seems to be independent of cholesterol level [1,14]. The mechanisms by which statins inhibit inflammation are not fully understood. It has been reported that they suppress production of monocyte chemotactic protein-1 (MCP-1) [15], as well as matrix metalloproteinases (MMPs) [5,16,17]. Statins also decrease macrophage expression of soluble ICAM-1 and secretion of IL-1, IL-6, TNF-alpha [5,18-23].

A number of large clinical trials have been designed to investigate the effect of statins in treating acute coronary syndromes (ACS) [24-30] Because patients in these studies were randomized later after being admitted to hospital, often after they had been clinically stabilized, these randomized, double blind trials focused more on early secondary prevention, as opposed to evaluating earlier therapy to target plaque instability in ACS. Furthermore, broad exclusion criteria in some of these trials, including coronary intervention during the index hospitalization visit, deter from the generalizability of their results to the majority of patients treated according to current clinical practice [24]. Nevertheless, these studies have shown promising results, despite the fact that the statins were administered after activation of the immune mechanisms was completed and after the inflammatory reaction was already fully developed.

**Figure 1:**
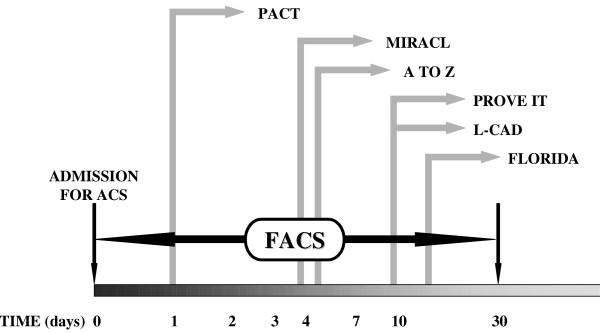
Design of the FACS trial in comparison to other trials evaluating statins in ACS patients. In the Pravastatin in Acute Coronary Treatment (PACT) trial statin, therapy was initiated within 24 hours of onset of ACS. In the Myocardial Ischemia Reduction With Aggressive Cholesterol Lowering (MIRACL) trial, patients were randomized 24 to 96 hours after ACS. In the Z-phase of the A-to-Z trial, simvastatin therapy was initiated within 5 days of the onset of ACS, after clinical stabilization. In the Pravastatin or Atorvastatin Evaluation and Infection Therapy (PROVE IT) trial, patients were randomized up to 10 days after ACS. In the Lipid-Coronary Artery Disease (L-CAD) trial, statin therapy was initiated up to ten days following the onset of ACS. In the Fluvastatin on Risk Diminishing After Acute Myocardial Infarction (FLORIDA) trial, patients were randomized up to two weeks after ACS.

Little information is available on the efficacy of statins in treating ACS at an earlier phase, i.e., at the time of hospital admission. Recently published data from experimental projects [31-35] and from small clinical trials [36-38]) have shown a positive effect of statins when they are administered in the acute phase of ACS. Our preliminary results with cerivastatin treatment in patients with non-ST segment elevation ACS starting at the time of hospital admission have shown the safety of such a strategy as well as a decrease in inflammatory markers (CRP, IL-6) by 24-hour follow-up, as compared to the non-treated group [39]. Based on these pilot data, we are conducting a clinical trial to evaluate fluvastatin therapy administered to patients with ACS immediately at the time of admission (Figure 1).

## Methods

### Objectives

The objectives of the FACS trial are to determine:

(i) Whether initiation of fluvastatin therapy in patients with ACS immediately after hospital admission decreases levels of CRP, IL-6, and pregnancy-associated plasma protein A/ proform eosinophilic major basic protein (PAPP-A/proMBP), which represent indirect markers of plaque instability and indicators of poor prognosis; and

(ii) Whether initiation of fluvastatin therapy decreases the occurrence of ischemic events (death, nonfatal myocardial infarction, recurrent symptomatic ischemia, urgent revascularization, cardiac arrest) in patients with ACS.

### Overview

This is a prospective, 30-day, multicenter, randomized, double-blind, placebo-controlled study in 1,000 patients with ACS. Patients are enrolled from 10 sites in the Czech Republic and Slovakia. At each institution, the protocol and the informed consent form are reviewed and approved by the institutional ethics committee before study initiation. Eligible patients are randomized to one of two treatment groups immediately after hospital admission (within one hour). One group is assigned 80 mg/day fluvastatin (Lescol XL), with the other group receiving placebo. Participants are followed on an intention-to-treat basis. The primary endpoint relates to levels of CRP, IL-6, and PAPP-A/proMBP. The secondary endpoint is the occurrence of an ischemic event, defined as death, nonfatal myocardial infarction (MI), recurrent symptomatic myocardial ischemia, cardiac arrest with resuscitation, and urgent revascularization.

### Study population

This study will enroll high-risk patients admitted to the hospital for ACS. Eligible patients with ST elevation ACS must have resting chest pain less than 12 hours before admission and either ≥ 1 mm ST-segment elevation in 2 or more continuous leads or new left bundle branch block on ECG. Those with non-ST elevation ACS must have resting chest pain during the previous 48 hours and either ≥ 1 mm ST segment depression or negative T waves in 2 or more continuous leads.

#### Exclusion criteria

Subjects are excluded from study participation if they are <18 years of age or if they have concomitant active liver disease or persistent elevation of transaminases (> 3 times the upper limit of normal), a history of lipid-lowering therapy less than 30 days before the index event or a known allergy to fluvastatin or to any additives present in the drug. Other exclusions include inability to ingest oral medication, unwillingness to be followed for the duration of the study, muscle disease (e.g., myositis), and creatine kinase ≥ 5 times the upper limit of normal due to conditions other than myocardial infarction. Women of childbearing potential who are pregnant, nursing or who are not using effective contraception will also be excluded.

### Follow-up

**Figure 2:**
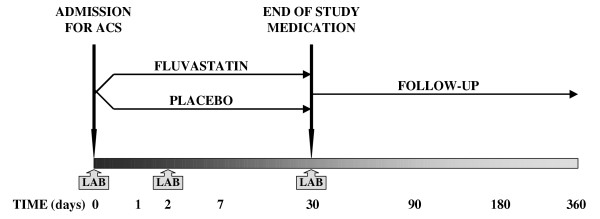
Study design of the FACS Trial. Patients admitted with acute coronary syndrome (ACS) are randomized to either fluvastatin 80 mg or placebo for 30 days. Patients are then followed for one year. Assessments of CRP, IL-6, and PAPP-A/proMBP (LAB) are performed at admission, on day 2, and day 30.

After obtaining informed consent, blood samples are taken from patients for examination of inflammatory markers (CRP, IL-6, and PAPP-A/proMBP). Patients are then randomized to 80 mg fluvastatin (Lescol XL) or to placebo immediately p.o. Medical history and physical examination, standard 12-lead ECG, blood lipid profile, and liver function tests are performed as part of participants' routine admission care. Fluvastatin 80 mg or placebo are then taken once daily for 30 days. Follow-up measurement of inflammatory markers (CRP, IL-6, and PAPP-A/proMBP) is performed on day 2 and day 30. Follow-up visits are scheduled at pre-discharge, day 30, 90, 180 and 360. Blood liver function and creatine kinase tests are done at pre-discharge and at the 30-day visits. At day 30, the lipid profile is also examined, and study medication is withdrawn. All visits include assessment of ischemic events and recent medical history since the last follow-up visit, including use of concomitant medications (Figure 2).

During follow-up, no specific recommendations are made with respect to diagnostic and therapeutic strategy, except that other lipid-lowering drugs should not be given after randomization until day 30. All management decisions are left to the discretion of each patient's treating physician.

### Safety

The principal safety concerns are hepatic dysfunction and myopathy. If a patient's serum transaminase levels are persistently elevated to > 3 times the upper limit of normal, the study medication is discontinued. Similarly, study medication is stopped if the patient develops muscle pain, weakness, or tenderness in association with a serum creatine kinase level > 10 times the upper limit of normal.

### Sample size

The trial will enroll 1,000 patients, to ensure adequate power to detect significant treatment benefit of 80 mg fluvastatin (Lescol XL) with respect to the primary endpoint (30-day decrease of CRP and IL-6) and the combined secondary endpoint (death, nonfatal myocardial infarction, recurrent symptomatic ischemia, urgent revascularization, cardiac arrest). With 500 patients randomized to 80 mg fluvastatin (Lescol XL) and 500 patients randomized to placebo, the trial will have more than 80 % power to detect a decrease in CRP level by 1.36 μg/L and a decrease in IL-6 level by 1.09 ng/L. Calculations are based on a two-sample *t*-test. The estimated combined secondary endpoint rate at 30-days is 20 %. Based on comparison of proportions with *p *= 0.05 test significance, the trial will have more than 80 % power to detect a 33% decrease in the combined secondary endpoint.

## Conclusion

The FACS trial is the first multicenter, randomized, double-blind, placebo-controlled trial investigating the effects of fluvastatin therapy started immediately after hospital admission in patients with ACS.

## Competing interests

The author(s) declare that they have no competing interests.

## Authors' contributions

PO has made substantial contributions to the concept and design of the trial and has drafted the manuscript. DA, PH, JVej, JK, MMat, and JVes have made substantial contributions to the concept and design of the study and have been involved in revising the manuscript. PB, MW, OA, JS, EN, FH, MR, and JC have been involved in the acquisition and analysis of data. MMac and MK have given final approval of the version to be published.
